# Partial substitution of red or processed meat with plant-based foods and the risk of type 2 diabetes

**DOI:** 10.1038/s41598-023-32859-z

**Published:** 2023-04-11

**Authors:** Mirkka Maukonen, Kennet Harald, Niina E. Kaartinen, Heli Tapanainen, Demetrius Albanes, Johan Eriksson, Tommi Härkänen, Pekka Jousilahti, Seppo Koskinen, Essi Päivärinta, Tiina Suikki, Hanna Tolonen, Anne-Maria Pajari, Satu Männistö

**Affiliations:** 1 Finnish Institute for Health and Welfare (THL), Mannerheimintie 166, PL 30, 00271 Helsinki, Finland; 2 National Cancer Institute, NIH, Bethesda, MD USA; 3 Department of General Practice and Primary Health Care, University of Helsinki and Helsinki University Hospital, 00014 Helsinki, Finland; 4 Folkhälsan Research Center, University of Helsinki, 00014 Helsinki, Finland; 5 Department of Obstetrics and Gynecology and Human Potential Translational Research Programme, Yong Loo Lin School of Medicine, National University of Singapore, Singapore, 119228 Singapore; 6 Singapore Institute for Clinical Sciences (SICS), Agency for Science, Technology and Research, (A*STAR), Singapore, Singapore; 7 University of Helsinki, Helsinki, Finland

**Keywords:** Risk factors, Diseases, Endocrine system and metabolic diseases, Type 2 diabetes, Epidemiology

## Abstract

High consumption of red and processed meat has been associated with increased type 2 diabetes (T2D) risk. These kinds of diets are also environmentally unsustainable. We examined a modeled association between a partial substitution of red meat or processed meat with plant-based foods (legumes, vegetables, fruit, cereals, or a combination of these) and T2D risk among Finnish adults. We used pooled data from five Finnish cohorts (n = 41,662, 22% women, aged ≥ 25 years, 10.9 years median follow-up with 1750 incident T2D cases). Diet was assessed by a validated food frequency questionnaire. In the substitution models, 100 g/week of red meat or 50 g/week of processed meat were substituted with similar amounts of plant-based substitutes. Cohort-specific hazard ratios (HRs) were estimated by Cox proportional hazards multivariable model and pooled using a two-staged random-effects model. We observed small, but statistically significant, reductions in T2D risk in men when red or processed meat were partially substituted with fruits (red meat: HR 0.98, 95% CI 0.97–1.00, *P* = 0.049, processed meat: 0.99, 0.98–1.00, *P* = 0.005), cereals (red meat: 0.97, 0.95–0.99, *P* = 0.005, processed meat: 0.99, 0.98–1.00, *P* = 0.004) or combination of plant-based foods (only processed meat: 0.99, 0.98–1.00, *P* = 0.004) but not with legumes or vegetables. The findings of women were similar but not statistically significant. Our findings suggest that even small, easily implemented, shifts towards more sustainable diets may reduce T2D risk particularly in men.

## Introduction

Diabetes is a significant public health concern, and its prevalence has been steadily increasing globally over the past decades. In 2019, approximately 463 million adults were living with diabetes and the prevalence is projected to reach 10.9% (700 million) by 2045^1^. Also, in Finland the total number of people living with type 2 diabetes (T2D) has been increasing over the past decades^2^. Diet is an important modifiable risk factor for T2D along with obesity and physical activity^3,4^.

Evidence from cohort studies have shown that high red and processed meat consumption is associated with higher T2D risk, whereas fiber rich diets have been found to reduce the risk^4,5^. In addition to health benefits, diets low in red and processed meat and high in plant-based foods have been found to be more environmentally sustainable^6–8^. Overall meat consumption is on the rise globally due to increasing average household incomes and due to population growth^7^. In Finland, the diets of adults have generally improved during the past 20 years but, according to the most recent dietary survey from 2017, they are still far from the current nutrition recommendations as nearly 80% of men and 26% of women exceeded the recommendation of red and processed meat consumption (500 g/week), whereas regarding vegetables, fruits, and berries only 14% of men and 22% of women reached the recommended minimum intake (≥ 500 g/week)^9,10^. Thus, there is a need for a shift from animal-based diets to more healthy and environmentally sustainable diets with more plant-based foods. A partial moderate substitution of red and processed meat with plant-based foods such as legumes, vegetables, nuts, fruits, and cereals could promote this move.

No previous study has focused on plant-based substitutes or has included vegetables or fruits in their meat substitution models on T2D risk. However, two studies based on the same three cohorts (Health Professionals Follow-up Study (HPFS), Nurses’ Health Study (NHS), and Nurses’ Health Study II (NHS II)) showed that a modeled partial substitution of red and processed meat with legumes^11^, cereals^12^, or nuts^11,12^ was associated with lower T2D risk. A study based on the European Prospective Investigation into Cancer (EPIC) data found similar results when red and processed meat were partially substituted with cereals or nuts but not with legumes^13^. In the Danish Diet, Cancer, and Health cohort, no association was found when red and processed meat were partially substituted by whole grains or refined grains^14^. In these studies, the substituted amounts of red and processed meat ranged between 50 and 100 g/day making up to 700 g/week. However, more subtle substitutions could be more encouraging for individuals to make changes in their diets and eventually result in more permanent and effective dietary changes in real-life settings.

Therefore, our aim was to study whether a moderate partial substitution of red meat (100 g/week) or processed meat (50 g/week) with similar amounts of plant-based foods (legumes, vegetables, fruits, cereals, or a combination of these) reduced T2D risk among Finnish adults.

## Methods

This study included participants from five Finnish cohorts: Alpha-Tocopherol, Beta-Carotene Cancer Prevention Study (ATBC) including smoking men^15^, Health 2000 Survey (Health 2000)^16^, Helsinki Birth Cohort Study (HBCS)^17^, DIetary, Lifestyle, and Genetic determinants of Obesity and Metabolic syndrome 2007 Study (DILGOM 2007)^18^ and the National FINRISK 2012 Study^19^ (Table 1). Each cohort included a health examination (including health measures and collection and analysis of blood samples) and self-administered questionnaires (including a food frequency questionnaire (FFQ)) and were linked to the national health registries. After the exclusion of prevalent T2D cases at baseline (n = 2925), our final data consisted of 41,662 men and women aged 25 years and over who had an acceptable FFQ.

**Table 1:** Cohorts included in the pooled analyses of partial substitution red or processed meat with plant-based foods and type 2 diabetes risk.

Cohorts	Baseline years	Baseline age, range	Final number of participants^1^, n	Men, n (%)	Follow-up time, median years	Type 2 diabetes, incident cases, n
Alpha-Tocopherol, Beta-Carotene Cancer Prevention Study (ATBC)^2^	1984–1988	50–70	25,342	25,342 (100%)	10.9	654
Health 2000 Survey (Health 2000)^3^	2000–2001	30–99	5695	2517 (44%)	15.2	608
Helsinki Birth Cohort Study (HBCS)^4^	2001–2004	56–69	1938	896 (46%)	11.1	70
DIetary, Lifestyle, and Genetic determinants of Obesity and Metabolic syndrome 2007 Study (DILGOM 2007)^5^	2007	25–74	4447	2015 (45%)	10.8	308
National FINRISK 2012 Study (FINRISK 2012)^6^	2012	25–74	4240	1874 (44%)	5.8	110
Total	–	–	41,662	32,644 (78%)	10.9	1750

^1^Including participants with an acceptable food frequency questionnaire (FFQ) and without prevalent type 2 diabetes at baseline.

^2^The ATBC Cancer Prevention Study Group 1994^13^.

^3^Heistaro 2008^14^.

^4^Eriksson 2011^15^.

^5^Konttinen et al. 2010^16^.

^6^Borodulin et al. 2018^17^.

This study was performed in line with the principles of the Declaration of Helsinki and each cohort study followed the current code of ethics at the time of the study. For example, in the more recent cohorts, the Ethics Committee of Helsinki and Uusimaa Hospital District approved the research protocols. All participants signed an informed consent.

### Dietary assessment

In all cohorts, the habitual diet over the last 12 months was assessed by a widely used and validated FFQ developed and regularly updated at the Finnish Institute for Health and Welfare (THL)^20–23^. In the ATBC study, FFQs were filled at home and the quality of FFQ was checked together with a trained study nurse during the health examination (reserved time for the FFQ checking was 30 min). In the other cohorts, FFQs were either completed at the study site (HBCS, DILGOM 2007) or at home (HEALTH 2000, FINRISK 2012) and then sent by mail to the THL. At THL the quality of FFQs was checked and incompletely filled FFQs (blank questionnaires or sections, questionnaires with several blank rows) were excluded. Furthermore, exclusion has been made on FFQs reporting extreme and implausible energy intake values at both ends of the energy intake distributions based either on daily energy intake cut-offs corresponding to 0.5%^24^ at both ends of sex-specific daily energy intake distributions (HBCS, DILGOM 2007, FINRISK 2012) or daily energy intake values < 600 and > 7000 kcal/day (Health 2000). The average daily food consumption and energy intake were calculated using in-house software and the Finnish National Food Composition Database (Fineli®)^25^.

In our substitution models, we partially substituted red meat or processed meat with plant-based foods (legumes, vegetables, fruits, cereals, or a combination of these). We examined red and processed meat separately because processed meat has been more consistently associated with lower T2D risk than red meat^5^. Red meat included beef, pork, lamb, and game. Processed meat included sausages and cold cuts. Legumes included all forms of commonly used legumes in Finland (green peas, green beans, beans, and soya). Vegetables included all vegetables (excluding legumes) along with nuts, seeds and almonds of which consumption is in general low in the Finnish population^9^. Fruits included all fruits and berries. Cereals included rye, oat, barley, and wheat. The meat substitutions were considered as 100 g/week for red meat and 50 g/week for processed meat reflecting common daily consumption of red meat or processed meat. The plant-based food substitutes were also considered either as 100 g/week or 50 g/week depending on which meat they substitute. These amounts of plant-based foods are also within the recommended serving sizes of the current Finnish nutrition recommendations for legumes, vegetables, and fruits or within the recommended serving size or daily intake for cereals depending on consumed cereal food^26^.

### Type 2 diabetes ascertainment

T2D cases were identified by linking participants to the nationwide administrative registries on reimbursement for diabetes medication expenses (except the HBCS), medication purchases (Anatomical Therapeutic Chemical (ATC) codes beginning with A10) (except the ATBC and the HBCS), hospitalizations (except the ATBC) or cause of death (International Classification of Diseases, Tenth Revision (ICD10) codes E11-14) (except the ATBC). Participants were linked to the registries through a unique personal identity code assigned to each Finnish citizen. The first registered date was used as the date of first event. In Finland, patients with medical treatment for diabetes are entitled to reimbursement of their medication expenses according to sickness insurance legislation. In order to get reimbursement, a medical certificate describing the diagnostic criteria applied for T2D diagnosis is required from the attending physician. The certificate is then verified to fulfill the diagnostic criteria at the Social Insurance Institution (Kela), which maintains a central register of all persons prescribed and receiving reimbursement for medicine expenses.

### Potential confounding variables

The self-administered questionnaires included questions on socioeconomic status, lifestyle factors, and medical history, for example. These variables were harmonized across the cohorts. Education was categorized into sex and birth cohort specific thirds (low, middle, high) except for the ATBC-study, where ‘highest education’ included those with more than an upper secondary education. Participants’ smoking habits were categorized into three classes (never, former, or current smoker) (the ATBC-study included only current smokers). Leisure time physical activity was categorized into three classes: passive (light activities such as reading), somewhat active (e.g., walking or gardening) or active (competition or other heavy sports). Hormone replacement therapy (women) was categorized as: ever (current or former user) or never (never used). At study sites, trained study nurses measured diastolic (mmHg) and systolic blood pressure (mmHg), weight and height and took blood samples of participants according to standard protocols^27^. Body mass index (BMI) was calculated as a participant’s weight in kilograms divided by the square of height in meters (kg/m^2^). Serum total cholesterol (mmol/l) was analyzed from blood samples.

### Statistical analysis

Substitution analyses were conducted separately for men and women because in the Finnish diet in general, there are sex-related differences in the consumption of most foods considered central in this study^9,10^. Baseline characteristics of the study participants by cohorts are reported as medians with interquartile ranges (IQR) for continuous variables and as proportions for categorical variables.

In the substitution analyses we used a two-staged random-effects model with Cox proportional hazards multivariable model where pooled hazard ratios (HR) and corresponding 95% CI were estimated from the cohort-specific HRs weighted by the inverse of their variance^28^. The heterogeneity between the pooled cohorts were tested by Q-statistics. The substitution analyses were based on the leave-one-out model in which the substitution models included the substitute (legumes, vegetables, fruits, cereals, or a combination of these) as a separate variable and a sum variable including the substitute and the substituted food group (red meat or processed meat)^29,30^. The derived HRs of the substitutes were interpreted as the estimated T2D risk related to increased intake of each plant-based substitute and a concurrent decrease in intake of the substituted meat groups. For red meat, the decrease in intake considered in substitution models was 100 g/week and for processed meat 50 g/week with similar increases in intakes of the plant-based substitutes. In case of significant associations, we ran additional analysis. First, we conducted the substitution analysis by doubling the substituted amounts so that 200 g/week of red meat and 100 g/week of processed meat were substituted with similar amounts of the significantly associated plant-based substitutes. Secondly, we conducted the substitution analysis by stratifying the data according to the tertiles of baseline consumption levels of the significantly associated plant-based foods to examine whether the magnitude of associations differed according to the baseline consumption levels.

Furthermore, regarding cereals, analyses were conducted also without wheat since whole grains have been associated with lower T2D risk (e.g.^31^) and we were not able to separate whole wheat from refined wheat. For sensitivity analyses, we also excluded those with lower than 100 g/week consumption of red meat or lower than 50 g/week of processed meat from the analyses. Additionally, substitution analyses were conducted by excluding T2D cases diagnosed during the first two years of follow-up, due to concerns about reverse causation.

Analyses were controlled for potential confounding factors based on the literature. First, we adjusted for age and energy intake (model 1). The second model (model 2) was further adjusted for education, smoking, BMI, leisure-time physical activity, diastolic blood pressure, systolic blood pressure, total serum cholesterol, hormone replacement therapy (women), alcohol intake (as ethanol) and consumption of sugar sweetened beverages, dairy, and coffee.

Statistical analyses were performed using R statistical software, version 3.6.3^32^ and pooling of hazard ratios was done using R package meta^33^.

## Results

The pooled data included 41,662 men and women (aged ≥ 25 years, 22% of women) with 1750 incident T2D cases over the 10.9 years (range 5.8–15.2 years) median follow-up time (Table 1).

At baseline, the median age of the participants ranged from 50 (Health 2000) to 60 years (HBCS) (Table 2). Participants of the ATBC study were all men and current smokers due to the study design. In addition, participants of the ATBC study were less likely to have high education and to be physically active in their leisure time compared to the other cohorts. The median BMI ranged from 25.9 kg/m^2^ (ATBC, DILGOM 2007) to 26.9 kg/m^2^ (HBCS) across the cohorts. Women in the HBCS study more likely used hormone replacement therapy (68%) than women in the other cohorts (14–31%). Regarding food groups, the ATBC study seemed to differ the most from the other cohorts. For example, regarding substitution foods participants of the ATBC study tended to have higher consumption of processed meat and cereals and lower consumption of legumes, vegetables, and fruits. Furthermore, there also seemed to be differences between sexes so that men compared to women tended to have higher consumption of red meat and processed meat, legumes and cereals and lower consumption of vegetables and fruits in each cohort (data not shown).

**Table 2:** Medians (interquartile ranges (IQR)) or percentages of characteristics of participants included in the pooled analysis by cohorts.

	ATBC	Health 2000	HBCS	DILGOM 2007	FINRISK 2012
	All (men 100%)	All	All	All	All
Confounding factors					
Age, years	57.1 (8.0)	50.0 (22.0)	60.0 (4.0)	52.1 (21.7)	52.0 (25.0)
High education^1^, %	6	34	33	36	34
Current smoker, %	100	26	24	18	17
Active in leisure time, %	6	17	26	29	32
Body mass index, kg/m^2^	25.9 (4.7)	26.2 (5.8)	26.9 (5.4)	25.9 (5.5)	25.9 (5.7)
Systolic blood pressure, mmHg	140 (26)	131 (28)	144 (27)	131 (25)	130 (23)
Diastolic blood pressure, mmHg	88 (14)	81 (15)	88 (14)	79 (15)	80 (15)
Total serum cholesterol, mmol/l	6.2 (1.5)	5.9 (1.4)	5.9 (1.4)	5.2 (1.3)	5.3 (1.4)
Hormone replacement therapy (women), ever %	–	31	68	16	14
Energy, MJ/day	10,866 (4017)	9074 (3907)	8696 (4038)	9866 (4529)	8883 (4100)
Alcohol, g/day	11 (23)	2 (7)	5 (11)	4 (9)	4 (9)
Sugar sweetened beverages, g/day	57 (168)	77 (164)	52 (160)	86 (178)	73 (159)
Dairy, g/day	697 (503)	546 (469)	437 (433)	581 (510)	581 (517)
Coffee, g/day	550 (350)	375 (323)	375 (235)	375 (461)	500 (550)
Substitution variables					
Red meat, g/day	65 (39)	73 (49)	59 (50)	73 (58)	65 (51)
Processed meat, g/day	60 (57)	36 (48)	30 (39)	40 (49)	37 (47)
Legumes, g/day	4 (5)	9 (9)	8 (9)	10 (9)	10 (10)
Vegetables^2^ (excl. legumes), g/day	94 (83)	217 (185)	245 (203)	263 (214)	227 (185)
Fruits, g/day	107 (116)	158 (206)	217 (273)	214 (239)	154 (182)
Cereals, g/day	205 (109)	133 (81)	121 (72)	155 (92)	127 (87)

^1^The total number of school years was divided into birth cohort specific tertiles with the exception of the ATBC study, where ‘highest education’ included those with more than upper secondary education.

^2^Nuts and seeds are included in vegetables.

Both red meat and processed meat were positively associated with T2D risk, but the finding was statistically significant only for processed meat (*P*_trend_ = 0.010, model 2) (Supplemental Table S1). Small increases in risks were also found with the amounts used in our substitution analysis (red meat (100 g/week): HR 1.01, 95% CI 1.00–1.03, *P* = 0.041, processed meat (50 g/week): 1.01, 1.00–1.01, *P* = 0.006, model 2). No associations were found regarding the plant-based substitutes, except for the positive association found between vegetables and T2D (*P*_trend_ = 0.034, model 2). These associations were in general similar in both sexes but slightly stronger in men (data not shown).

In men, we found small but statistically significant reductions in T2D risk when 100 g/week of red meat or 50 g/week of processed meat were substituted with similar amounts of vegetables (only processed meat), fruits, cereals, or the combination of plant-based foods (model 1, Table 3). After further adjustments (model 2) red meat substitutions with fruits (0.98, 0.97–1.00, *P* = 0.049) or cereals (0.97, 0.95–0.99, *P* = 0.005) remained statistically significant but the substitution with combination of plant-based foods attenuated to non-significant (*P* = 0.066). Regarding processed meat, the substitutions with fruits (0.99, 0.98–1.00, *P* = 0.005), cereals (0.99, 0.98–1.00, *P* = 0.004) or the combination of plant-based foods (0.99, 0.98–1.00, *P* = 0.004) remained statistically significant but the substitution with vegetables attenuated to non-significant (*P* = 0.11). Substitutions of red or processed meat with legumes resulted in no significant associations in either model. In women, partial substitution of red or processed meat with vegetables, fruits, cereals, and the combination of plant-based foods similarly suggested lower T2D risk (model 1, Table 3). Further adjustments (model 2), however, attenuated these associations to non-significant.

**Table 3:** Pooled associations between partial substitutions of red meat or processed meat with legumes, vegetables, fruits, cereals or the combination of plant-based foods and type 2 diabetes risk in men and women.

	Men			Women		
	HR (95% CI) Model1	HR (95% CI) Model2	*P*_het._^1^	HR (95% CI) Model1	HR (95% CI), % Model2	*P*_het._^1^
Substitution of red meat (100 g/week) with						
Legumes, 100 g/week	1.02 (0.92, 1.14)	1.05 (0.95, 1.16)	0.11	1.02 (0.93, 1.12)	1.01 (0.90, 1.14)	0.32
Vegetables^2^ (excluding legumes), 100 g/week	0.98 (0.96, 1.00)	0.99 (0.97, 1.01)	0.50	0.96 (0.94, 0.99)*	0.99 (0.96, 1.02)	0.53
Fruits, 100 g/week	0.98 (0.96, 0.99)*	0.98 (0.97, 1.00)*	0.56	0.96 (0.93, 0.99)*	0.99 (0.96, 1.01)	0.50
Cereals, 100 g/week	0.96 (0.93, 0.99)*	0.97 (0.95, 0.99)*	0.58	0.95 (0.92, 0.98)*	0.99 (0.95, 1.02)	0.96
Legumes, vegetables, cereals, and fruits, 100 g/week	0.98 (0.96, 1.00)*	0.98 (0.97, 1.00)	0.43	0.96 (0.93, 0.99)*	0.98 (0.96, 1.01)	0.58
Substitution of processed meat (50 g/week) with						
Legumes, 50 g/week	1.01 (0.97, 1.05)	1.02 (0.98, 1.06)	0.25	1.02 (0.98, 1.07)	1.03 (0.99, 1.08)	0.50
Vegetables^2^ (excluding legumes), 50 g/week	0.98 (0.98, 0.99)*	0.99 (0.99, 1.00)	0.86	0.98 (0.97, 0.99)*	1.00 (0.98, 1.02)	0.57
Fruits, 50 g/week	0.98 (0.97, 0.99)*	0.99 (0.98, 1.00)*	0.95	0.98 (0.97, 0.99)*	1.00 (0.98, 1.02)	0.63
Cereals, 50 g/week	0.97 (0.95, 0.99)*	0.99 (0.98, 1.00)*	0.52	0.97 (0.95, 0.99)*	1.00 (0.98, 1.03)	0.25
Legumes, vegetables, cereals, and fruits, 50 g/week	0.98 (0.96, 0.99)*	0.99 (0.98, 1.00)*	0.93	0.98 (0.96, 0.99)*	1.00 (0.98, 1.01)	0.54

Model 1: adjusted for age (years, continuous) and energy (kJ/day, continuous).

Model 2: adjusted for Model 1 + education (tertiles by birth year), smoking (never, former, current), body mass index.

(kg/m2, continuous), leisure-time physical activity (passive, somewhat active, active), diastolic blood pressure (mmHg, continuous), systolic blood pressure (mmHg, continuous), total serum cholesterol (mmol/l, continuous), hormone replacement therapy (women) (ever, never), alcohol (as ethanol, g/day, continuous), sugar sweetened beverages (g/day, continuous), dairy (g/day, continuous) and coffee (g/day, continuous).

*P < 0.05.

^1^*P* for heterogeneity from Q-statistics between pooled cohorts. Adjusted as Model 2.

^2^Nuts and seeds are included in vegetables.

No significant heterogeneities were shown between the cohorts in the association between partial substitutions and T2D risk in either sex (Table 3).

Regarding the significant associations found in men, we conducted further analyses by doubling the amounts so that 200 g/week of red meat was substituted by 200 g/week fruits or cereals, which resulted in slightly larger reductions in T2D risk (fruit: 0.97, 0.93–1.00, *P* = 0.049, cereal: 0.94, 0.90–0.98, *P* = 0.005, model 2). The same was seen when the amount of processed meat was doubled (100 g/week) (fruit: 0.98, 0.97–0.99, *P* = 0.005, cereal: 0.97, 0.96–0.99, *P* = 0.004, combination of plant-based foods: 0.98, 0.97–0.99, *P* = 0.004, model 2). When we conducted the substitution analysis by stratifying the data according to the tertiles of baseline consumption levels of cereals or fruits in men no significant changes in the magnitude of the results were observed between the highest and the lowest consumption tertiles of these foods (data not shown).

When substitution analyses regarding cereals were conducted by excluding wheat from cereals, the results remained similar in men (red meat: 0.97, 0.94–0.99, *P* = 0.006, processed meat: 0.98, 0.98–0.99, *P* = 0.002, model 2) and in women (red meat: 1.00, 0.96–1.05, *P* = 0.94, processed meat: 1.01, 0.98–1.04, *P* = 0.48, model 2) compared to the original results on cereals including wheat.

The results remained similar also when those with lower than 100 g/week consumption of red meat or lower than 50 g/week of processed meat were excluded from the substitution analyses or when those diagnosed with T2D during the first 2 years of follow-up were excluded (Supplemental Table S2).

## Discussion

In this large, pooled cohort of 41,662 Finnish adults, we studied whether a moderate partial substitution of red meat or processed meat with legumes, vegetables, fruits, cereals, or a combination of the plant-based foods was associated with a reduced risk of T2D. Our findings indicated that substituting red meat or processed meat with fruits, cereals, or combination of the plant-based foods (processed meat) slightly reduced the risk of T2D particularly in men. As for substitutions with legumes or vegetables, no associations were found in either sex.

Our findings suggested small reductions in T2D risk when 100 g/week of red meat (2% reduction in risk) or 50 g/week of processed meat (1% reduction in risk) were substituted with similar amounts of fruits in men. Findings were similar in women but after adjusting for BMI and other relevant socio-economic and lifestyle factors these associations no longer reached statistical significance. Previous substitution studies have not included fruits in their substitution models. However, our findings seem plausible as the evidence gathered by a recent umbrella review of meta-analyses of prospective studies on dietary factors and T2D risk indicated a protective role for apples, pears, and berry fruits, although regarding total fruit consumption no association was found^4^. Fiber and polyphenol content of fruits has been suggested as a potential mechanism behind the association^34,35^. These suggested mechanisms were partly supported by the recent umbrella review of meta-analyses on dietary factors and T2D risk in which association was found for total polyphenol intake but not for fruit fiber^4^. Another possible explanation could be related to better weight maintenance associated with high fruit consumption which could indirectly influence the development of T2D^36^.

Our findings also suggested small reductions in T2D risk when red meat (3% reduction in risk) or processed meat (1% reduction in risk) were partially substituted with cereals in men. Three previous studies have included cereal substitutes with the difference from our study that they all modeled much larger substitutions amounts^12–14^. In a US study based on the HPFS, NHS and NHS II cohorts (n = 202,157), partial modeled substitutions of red meat (100 g/day) or processed meat (50 g/day) with whole grains (32 g/day of bread or 200 g/day of cooked brown rice or cereals) reduced T2D risk by 24% for red meat and by 35% for processed meat^12^. In a European study based on the EPIC data (n = 26,460), modeled substitution of red and processed meat (50 g/day) with cereals (refined and whole grain) (30 g/day) resulted in an 8% lower T2D risk^13^. In a Danish study based on the Danish Diet, Cancer, and Health cohort (n = 39,437) partial substitution of red and processed meat (100 g/day) with whole grains (30 g/day) or refined grains (30 g/day) was not associated with T2D risk^14^.

There is strong evidence from cohort studies showing an association between whole grain consumption and lower risk of T2D^4,31,37^. Furthermore, high intake of whole grains has been associated with better insulin sensitivity and lower fasting insulin concentrations^37,38^. These associations may be mediated by the high fiber content of whole grains but also through different phytochemicals, vitamins, and minerals that whole grains have high contents^38,39^. In the present study, the cereal variable included rye, oat, barley, and wheat and of these rye, oat and barley are mostly whole grains as for wheat we were unable to separate whole wheat from refined wheat. However, exclusion of wheat from the cereal analyses did not alter the findings.

Furthermore, we found that partial substitution of processed meat with combination of plant-based foods may reduce T2D risk in men. This association is most likely mainly explained by the previously mentioned significant associations found with fruit and cereal substitutions.

Regarding red or processed meat substitutions with legumes, no significant associations were found in either sex. As for vegetables, our findings suggested small reductions in T2D risk in women but after further adjustments the results attenuated to non-significant. Legume substitutes have been included in two previous studies^11,13^. A study based on HPFS, NHS, and NHS II (n = 148,853) showed that substituting one daily serving of red and processed meat with legumes was associated with 11% lower T2D risk^11^, whereas a study based on EPIC data (n = 26,460) found no association when red and processed meat (50 g/day) were substituted with legumes (50 g/day)^13^. Vegetables have not been included in the previous meat substitution studies, instead three studies included nuts (nuts were included in our vegetable variable). These studies reported lower T2D risk when red and processed meat were partially substituted with nuts^11–13^. However, in a recent umbrella review, consumption of vegetables, nuts, or legumes was not associated with T2D incidence^4^.

In general, our findings were similar in men and women, but they attenuated to non-significant in women after further adjustments. One explanation for the stronger associations occurring among men could be that men on average tend to have higher consumptions of red and processed meat and lower consumptions of plant-based foods than women and therefore the modeled substitution may yield stronger associations in men than women. Furthermore, another likely explanation could be that our pooled dataset included more men (78%) and, thus, the statistical power to detect associations among men was higher. Previous substitution studies have in general analyzed men and women together^11–14^.

Small but significant reductions in T2D risk were observed in our study even with small easily implemented substitutions amounts where substitutions were modeled on a weekly basis in contrast to the daily basis substitutions modeled in the previous meat substitution studies on T2D risk^11–14^. Our findings are encouraging since these kinds of small changes in diet are easier to achieve, maintain and thus more likely to become actual habits. Also, when these substituted amounts were doubled, the reductions in T2D risk were also increased. This was seen particularly when red meat (200 g/week) or processed meat (100 g/week) were substituted with cereals as the reductions in T2D risk in men increased from 3 to 6% for red meat and from 1 to 3%, for processed meat. The used amounts can also be translated into actual servings for public health purposes. Regarding meat, 100 g of red meat corresponds to approximately a small beef steak (cooked) and 50 g of processed meat correspond to a small frankfurter or 3–4 cold cuts slices, for example. For fruits 50–100 g correspond to 0.5–1 mandarins or 0.5–1 small apples, for example. For cereals, 50–100 g corresponds to 0.5–1 serving size of uncooked spaghetti (whole grain preferably), 2–5 slices of bread (whole grain preferably) or 1–2 large bowls of oatmeal, for example.

This study has some strengths and limitations. A large data combining five Finnish cohort studies, which included extensive information about participants’ background, lifestyle and health can be considered a strength. T2D cases were identified from national administrative registries; however, we were unable to capture those individuals with T2D having lifestyle treatment only or those with undiagnosed T2D. Also, the diagnostics of T2D have become more accurate over time and therefore there may be more undiagnosed T2D cases in the older cohorts compared to more recent ones. Furthermore, as each cohort was planned independently, several initial characteristics (e.g., age ranges and covariates) varied across the cohorts. Particularly the ATBC study including smoking men was different from the other cohorts. In this study we harmonized the classifications of covariates across the cohorts. Furthermore, the cohort-specific substitution analyses showed similar results across the cohorts with no notable heterogeneity between the cohorts. Thus, even though the ATBC study was the largest study including more homogenous participants compared to the other cohorts the findings were similar across the cohorts. In all five cohorts diet was assessed with validated FFQs^20–23^. FFQ is a widely used and acceptable method in epidemiological studies. However, as dietary assessment was based on self-report the possible recall or reporting biases cannot be ruled out (e.g., under- or over-reporting). We adjusted for energy intake in our analyses which corrects the biases related to reporting to some extent. Furthermore, diet and confounding factors were based on a single measure, and we were unable to examine changes over time. Thus, there might occur some misclassifications among these variables if they changed markedly during the follow-up period. However, excluding T2D cases diagnosed during the first 2 years of follow-up did not alter the findings and thus reverse causality affecting, for example, on eating habits is unlikely present. Also, it should be kept in mind that the modeled food substitutions were based on a statistical model rather than actual changes in diet and therefore our results may not fully be representative of real-life settings and that the substitutions may not be feasible during a single meal but rather to reflect longer-term changes in the habitual diet. Furthermore, since our modeled substitutions included foods with different energy contents (for example substitution of red meat with vegetables) it is likely that there remained some residual difference in energy intake (and other nutrients) between foods despite the models were adjusted for total energy intake^40^. This may need to be considered by other foods eaten. However, the substitutions modeled in the current study were very moderate (50–100 g /week) and otherwise the diets remained unchanged. Therefore, it is unlikely that these changes would have profound impact on the nutritional profiles of the individuals. It should also be noted that the health-conscious people are more likely to participate in health surveys which may have possibly produced bias toward the null.

In conclusion, our findings are in line with the previous literature suggesting that a partial substitution of red meat and processed meat with cereals may be associated with reduced risk of T2D. As a novel finding, our results also suggested similar associations for red meat or processed meat substitutions with fruits. These associations were particularly seen in men. These findings indicate that a shift towards more sustainable diets may also be beneficial in terms of T2D prevention.

## Supplementary Information

Supplementary Tables.

## Data Availability

The dataset used is available upon request through the Findata permit procedure. https://www.findata.fi/en/.
