# Supplementary material for: Partial substitution of red or processed meat with plant-based foods and the risk of type 2 diabetes
Source: Sci Rep. 2023 Apr 11;13:5874. doi: 10.1038/s41598-023-32859-z (PMC10090151; doi:10.1038/s41598-023-32859-z)
Supplement: Supplementary file 1 — Supplementary Tables. [file 41598_2023_32859_MOESM1_ESM.docx]

**Supplementary Information**

**Title:** Partial substitution of red or processed meat with plant-based foods and the risk of type 2 diabetes

**Journal**: Scientific Reports

**Authors**: Mirkka Maukonen, Kennet Harald, Niina E Kaartinen, Heli Tapanainen, Demetrius Albanes, Johan Eriksson, Tommi Härkänen, Pekka Jousilahti, Seppo Koskinen, Essi Päivärinta, Tiina Suikki, Hanna Tolonen, Anne-Maria Pajari, Satu Männistö

**Corresponding author**: Mirkka Maukonen, [mirkka.maukonen@thl.fi](mailto:mirkka.maukonen@thl.fi)

This file includes 2 supplemental tables. **Supplemental Table S1** describes the overall association between red or processed meat and plan-based substitutes with type 2 diabetes. **Supplemental Table S2** shows the results from the sensitivity analysis related to substitution analysis.

| Supplemental Table S1. Hazard ratios (HR) and confidence intervals (95% CI) for type 2 diabetes risk and the consumption of red or processed meat and the plant-based substitutes | | | | | | | | | |
| --- | --- | --- | --- | --- | --- | --- | --- | --- | --- |
|  |  | **Quintile 1** | **Quintile 3** | **Quintile 5** |  | **P_trend_** | **P_het. by study_²** |  | **HR (95% CI)**  **for 100¹ g/week** |
| Red meat |  |  |  |  |  |  |  |  |  |
| Median g/week (IQR) |  | 232 (97) | 456 (56) | 857 (286) |  |  |  |  |  |
| Type 2 diabetes, n |  | 322 | 311 | 454 |  |  |  |  |  |
| Model 1 |  | 1.00 | 1.04 (0.89, 1.22) | 1.29 (1.10, 1.52) |  | 0.002 | 0.96 |  | 1.03 (1.01, 1.04)* |
| Model 2 |  | 1.00 | 1.01 (0.76, 1.35) | 1.11 (0.91, 1.36) |  | 0.27 | 0.75 |  | 1.01 (1.00, 1.03)* |
| Processed meat |  |  |  |  |  |  |  |  |  |
| Median g/week (IQR) |  | 105 (71) | 356 (71) | 908 (417) |  |  |  |  |  |
| Type 2 diabetes, n |  | 333 | 317 | 371 |  |  |  |  |  |
| Model 1 |  | 1.00 | 1.32 (1.07,1.63) | 1.73 (1.35, 2.21) |  | <0.001 | 0.46 |  | 1.02 (1.02, 1.03)* |
| Model 2 |  | 1.00 | 1.11 (0.94, 1.31) | 1.17 (0.96, 1.43) |  | 0.010 | 0.66 |  | 1.01 (1.00, 1.01)* |
| Legumes |  |  |  |  |  |  |  |  |  |
| Median g/week (IQR) |  | 9 (11) | 38 (10) | 121 (86) |  |  |  |  |  |
| Type 2 diabetes, n |  | 290 | 331 | 517 |  |  |  |  |  |
| Model 1 |  | 1.00 | 0.91 (0.78, 1.08) | 0.95 (0.79, 1.15) |  | 0.69 | 0.48 |  | 1.06 (1.01, 1.11)* |
| Model 2 |  | 1.00 | 0.95 (0.81, 1.13) | 1.02 (0.76, 1.35) |  | 0.66 | 0.71 |  | 1.04 (0.99, 1.09) |
| Vegetables² |  |  |  |  |  |  |  |  |  |
| Median g/week (IQR) |  | 313 (165) | 913 (186) | 2481 (1232) |  |  |  |  |  |
| Type 2 diabetes, n |  | 235 | 342 | 539 |  |  |  |  |  |
| Model 1 |  | 1.00 | 1.00 (0.73, 1.35) | 0.87 (0.58, 1.32) |  | 0.36 | 0.20 |  | 1.00 (0.99, 1.01) |
| Model 2 |  | 1.00 | 1.19 (0.90, 1.57) | 1.36 (1.01, 1.85) |  | 0.034 | 0.36 |  | 1.00 (1.00, 1.01) |
| Fruits |  |  |  |  |  |  |  |  |  |
| Median g/week (IQR) |  | 215 (182) | 883 (188) | 2405 (1235) |  |  |  |  |  |
| Type 2 diabetes, n |  | 342 | 304 | 459 |  |  |  |  |  |
| Model 1 |  | 1.00 | 0.80 (0.67, 0.95) | 0.70 (0.48, 1.00) |  | 0.035 | 0.26 |  | 1.00 (0.99, 1.00)* |
| Model 2 |  | 1.00 | 0.98 (0.77, 1.25) | 0.99 (0.84, 1.17) |  | 1.00 | 0.51 |  | 1.00 (1.00, 1.01) |
| Cereals |  |  |  |  |  |  |  |  |  |
| Median g/week (IQR) |  | 614 (245) | 1237 (144) | 2113 (510) |  |  |  |  |  |
| Type 2 diabetes, n |  | 428 | 346 | 293 |  |  |  |  |  |
| Model 1 |  | 1.00 | 0.89 (0.67, 1.17) | 0.81 (0.49, 1.3 4) |  | 0.26 | 0.002 |  | 0.98 (0.95, 1.01) |
| Model 2 |  | 1.00 | 0.99 (0.83, 1.17) | 0.97 (0.76, 1.25) |  | 0.61 | 0.43 |  | 0.99 (0.98, 1.00) |
| IQR, interquartile ranges  Model 1: adjusted for age (years, continuous) and energy (kJ/day, continuous).  Model 2: adjusted for Model 1 + education (tertiles by birth year), smoking (never, former, current), body mass index (kg/m2, continuous), leisure-time physical activity (passive, somewhat active, active), diastolic blood pressure (mmHg, continuous), systolic blood pressure (mmHg, continuous), total serum cholesterol (mmol/l, continuous), hormone replacement therapy (women) (ever, never), alcohol (as ethanol, g/day, continuous), sugar sweetened beverages (g/day, continuous), dairy (g/day, continuous) and coffee (g/day, continuous).  **P*<0.05  ¹ 50 g for processed meat  ² Nuts and seeds are included in vegetables | | | | | | | | | |

**Supplemental Table S2.** Pooled associations between partial substitutions of red meat (100 g/week) or processed meat (50 g/week)

with legumes, vegetables, fruits and cereals and the risk of type 2 diabetes **in men and women**.

|  | **Men** | |  | **Women** | |
| --- | --- | --- | --- | --- | --- |
|  | Sensitivity analyses 1^1^  HR (95% CI) | Sensitivity analyses 2^1^  HR (95% CI) |  | Sensitivity analyses 1^1^  HR (95% CI) | Sensitivity analyses 2^1^  HR (95% CI) |
| **Substitution of red meat (100 g/week) with** |  |  |  |  |  |
| Legumes 100 g/week | 1.05 (0.95, 1.17) | 1.01 (0.93, 1.10) |  | 1.02 (0.92, 1.12) | 1.00 (0.88, 1.12) |
| Vegetables² (excluding legumes) 100 g/week | 0.99 (0.97, 1.01) | 0.99 (0.97, 1.01) |  | 0.98 (0.95, 1.01) | 0.99 (0.96, 1.02) |
| Fruits 100g/week | 0.99 (0.97, 1.00) | 0.98 (0.96, 1.00) |  | 0.98 (0.95, 1.01) | 0.99 (0.96, 1.02) |
| Cereals 100 g/week | 0.97 (0.95, 1.00)* | 0.97 (0.94, 0.99)* |  | 0.98 (0.95, 1.02) | 0.99 (0.95, 1.03) |
| Legumes, vegetables, cereals and fruits, 100 g/week | 0.99 (0.97, 1.01) | 0.98 (0.97, 1.00) |  | 0.98 (0.95, 1.01) | 0.99 (0.96, 1.02) |
|  |  |  |  |  |  |
| **Substitution of processed meat (50 g/week) with** |  |  |  |  |  |
| Legumes 50 g/week | 1.02 (0.98, 1.06) | 1.00 (0.96, 1.04) |  | 1.03 (0.98, 1.08) | 1.02 (0.96, 1.07) |
| Vegetables² (excluding legumes) 50 g/week | 0.99 (0.99, 1.00) | 0.99 (0.98, 1.00) |  | 1.00 (0.99, 1.02) | 1.01 (0.98, 1.05) |
| Fruits 50 g/week | 0.99 (0.98, 1.00)* | 0.99 (0.98, 1.00)* |  | 1.00 (0.99, 1.20) | 1.01 (0.98, 1.04) |
| Cereals 50 g/week | 0.99 (0.98, 1.00)* | 0.99 (0.98, 1.00)* |  | 1.00 (0.98, 1.03) | 1.02 (0.97, 1.07) |
| Legumes, vegetables, cereals and fruits, 50 g/week | 0.99 (0.98, 1.00)* | 0.99 (0.98, 1.00)* |  | 1.00 (0.98, 1.02) | 1.01 (0.98, 1.05) |

Sensitivity analyses 1: Excluding participants who consumed red meat ˂100 g/week (n=777) and processed meat ˂50 g/week (n=1600).

Sensitivity analyses 2 Excluding cases diagnosed during the first two years of follow-up, due to concerns about reverse causation: ATBC (n=18),

Health 2000 (n=57), Helsinki Birth Cohort (n=8), DILGOM 2007 (n=51) and FINRISK 2012 (n=38).

* *P*<0.05

^1^ Adjusted for age (years), energy (kJ/day, continuous), education (tertiles by birth year), smoking (never, former, current), body mass index

(kg/m2, continuous), leisure-time physical activity (passive, somewhat active, active), diastolic blood pressure (mmHg, continuous), systolic blood pressure

(mmHg, continuous), total serum cholesterol (mmol/l, continuous), hormone replacement therapy (women) (ever, never), alcohol (as ethanol, g/day, continuous),

sugar sweetened beverages (g/day, continuous), dairy (g/day, continuous) and coffee (g/day, continuous).

² Nuts and seeds are included in vegetables
